# Dynamics of Endoreplication during *Drosophila* Posterior Scutellar Macrochaete Development

**DOI:** 10.1371/journal.pone.0038714

**Published:** 2012-06-06

**Authors:** Akihito Kawamori, Kouhei Shimaji, Masamitsu Yamaguchi

**Affiliations:** 1 Department of Applied Biology, Kyoto Institute of Technology, Sakyo-ku, Kyoto, Japan; 2 Insect Biomedical Research Center, Kyoto Institute of Technology, Sakyo-ku, Kyoto, Japan; Baylor University, United States of America

## Abstract

Endoreplication is a variant type of DNA replication, consisting only of alternating G1 and S phases. Many types of *Drosophila* tissues undergo endoreplication. However, the timing and the extent to which a single endocycling macrochaete undergoes temporally programmed endoreplication during development are unclear. Here, we focused on the dynamics of endoreplication during posterior scutellar (pSC) macrochaete development. Quantitative analyses of C values in shaft cells and socket cells revealed a gradual rise from 8C and 4C at 8 hours after pupal formation (APF) to 72C and 24C at 29 hours APF, respectively. The validity of the values was further confirmed by the measurement of DNA content with a confocal laser microscope. BrdU incorporation assays demonstrated that shaft cells undergo four rounds of endoreplication from 18 to 29.5 hours APF. In contrast, socket cells undergo two rounds of endoreplication during the same period. Statistical analyses showed that the theoretical C values, based on BrdU assays, nearly coincide with the actually measured C values in socket cells, but not in shaft cells after 22 hours APF. These analyses suggest that socket cells undergo two rounds of endoreplication. However, the mechanism of endoreplication in the shaft cells may change from 22 hours APF, suggesting the possibility that shaft cells undergo two or four rounds of endoreplication during the periods. We also found that the timing of endoreplication differs, depending on the type of macrochaete. Moreover, endocycling in shaft cells of both the left and right sides of pSC bristle lineages occurs in the same pattern, indicating that the process is synchronized for specific types of macrochaete. Our findings suggest that endocycling in macrochaete cell lineages can be a model for understanding mechanisms of endoreplication at the single-cell level.

## Introduction

Endoreplication is a variant of DNA replication consisting of only G1 and S phases [1] which is an essential part of the normal development of various organisms [2]. Many differentiated cells in plants, insects and some mammalian cells utilize this type of cell cycle to increase cell mass and genomic DNA content [1], [2]. They also employ endoreplication as a part of terminal differentiation to provide the nutrients and proteins needed to support the developing egg or embryo, or to support a specialized function of differentiated cells [2]. Three types of cell cycle have been suggested to drive endoploidy [2]; endocycling, re-replication and endomitosis. A key feature of the endocycle is that DNA content increases by clearly delineated doubling [2]. By way of contrast, re-replication is characterized by uncontrolled, continuous re-initiation of DNA synthesis within a given S phase, resulting in increases in DNA content without clearly recognizable genome doubling [2]. Finally, during endomitosis the cell enters but does not complete mitosis resulting in replicated copies of the chromosomes being incorporated into the same nucleus [2]. Programmed endoreplication often leads to high levels of genome amplification. For example, endocycling salivary gland cells have up to 2,048 copies of the euchromatic genome neatly aligned in parallel arrays [1], [3], while megakaryocytes which enter endomitosis to produce platelets become polyploid up to 128 N (normal haploid chromosome number) [4].

Endoreplication differs from DNA replication in the mitotic cycle in several aspects. First, genomic DNA synthesis during S phase is incomplete leaving approximately 30% of the genome as under-replicated [1]. Second, the normal cell cycle is driven by periodic activation of S-phase CycE/CDK complexes [2], [5], [6]. By contrast, in most endocycling cells mitotic cyclins are neither expressed nor required [1], [7]–[12]. Third, because of the lack of mitosis during endocycles, cell growth and DNA replication are tightly associated. Moreover, endoreplication is more susceptible to the effects of external influences such as nutrients and endocrine hormones [1]. Finally, while many of the proteins required for DNA replication and the regulation of the G1–S program are shared between the mitotic cycle and the endocycle [1], [2], [9], components of pre-replicative complexes such as *disc proliferation abnormal* (*dpa* known as *mcm4*) and *orc1* genes are only required for DNA replication in the mitotic cycle [13]–[15], implying that the mechanism of endoreplication is rather different. For all these reasons, studies on the endocycle can provide insights into the regulatory principles underlying the once per cell cycle replication of the genome and the relationship between S phase and mitosis [9].

**Figure 1:**
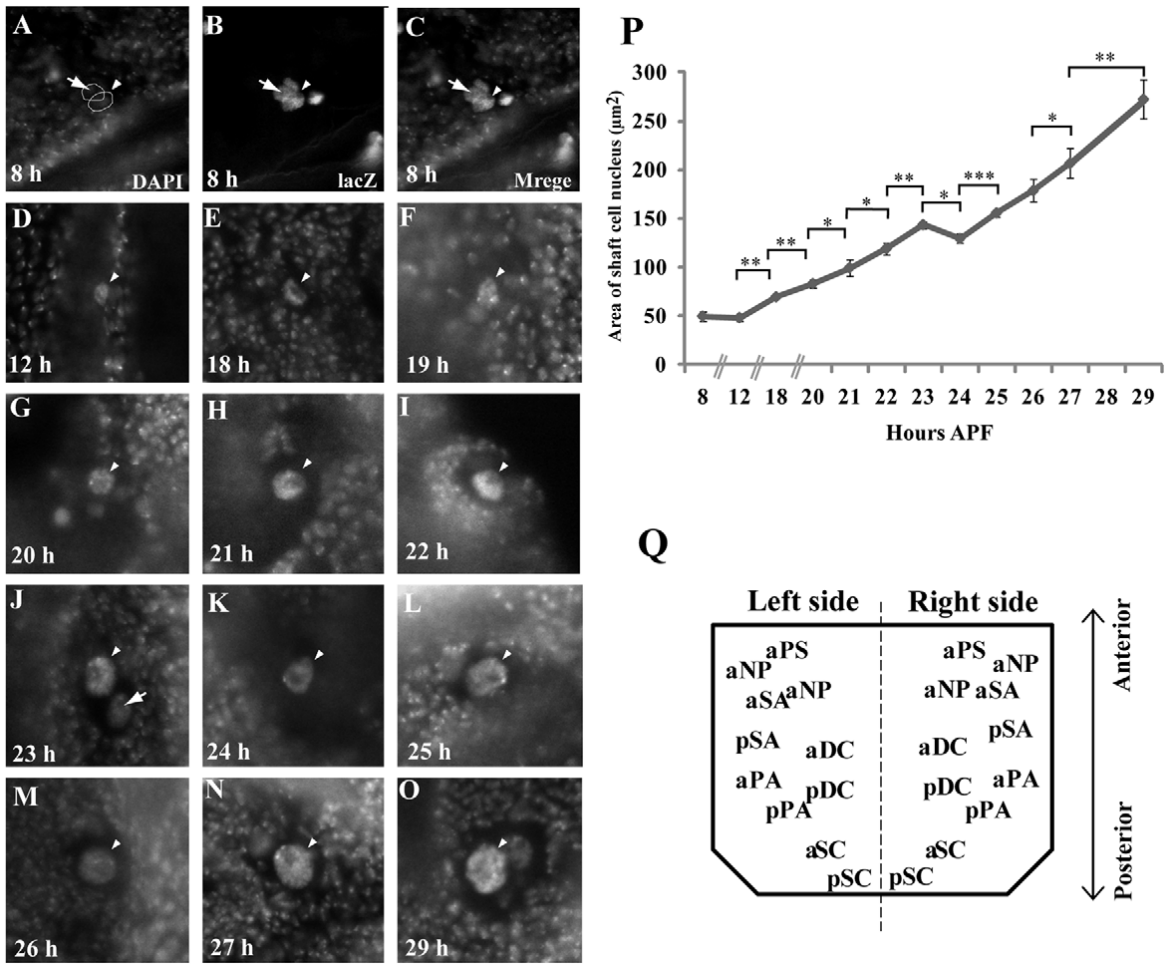
Dynamics of nuclear growth in pSC shaft cells from 8 to 29 hours APF. (A–O) Images of pSC shaft cells from 8 to 29 hours APF. Pupae with the *A101-lacZ /+* genotype were grown till 8(n = 6), 12(n = 3), 18(n = 8), 20(n = 11), 21(n = 8), 22(n = 8), 23(n = 3), 24(n = 7), 25(n = 5), 26(n = 11), 27(n = 6), 29 hours APF (n = 7) and dissected thoraxes were stained with anti-lacZ and DAPI. Arrows indicate socket cell nuclei and arrowheads shaft cell nuclei. (P) Dynamics of nuclear growth in a pSC shaft cell from 8 to 29 hours APF. The Y axis indicates the section area of the shaft cell nucleus. Significant differences in mean values at each time point to those of adjacent time points were set at *P<0.05, **P<0.01 and ***P<0.001. (Q) Illustration of 22 macrochaetes on left and right sides of an adult thorax. pSC, posterior scutellar; aSC, anterior scutellar; pDC, posterior dorsocentral; aDC, anterior dorsocentral; pPA, posterior postalar; aPA, anterior postalar; pSA, posterior supraalar; aSA, anterior supraalar; pNP, posterior notopleural;, aNP, anterior notopleural; PC, presutural.

In *Drosophila* the shaft and socket cells of sensory bristles, the macrochaetes and microchaetes utilize endocycling. The macrochaete is a type of large mechanosensory bristle on the *Drosophila* adult thorax which consists of four differentiated cells (shaft, socket, sheath and neuron). There are 11 macrochaetes (Fig. 1Q) and more than 200 microchaetes [16]. The differentiation of the sensory organ precursors (SOPs) of macrochaetes occurs within proneural clusters (PNCs) at the third instar larval stage [17]. The differentiated SOP of a bristle asymmetrically divides to produce a PIIa cell and a PIIb cell. The PIIa cell divides to give rise to a shaft cell and a socket cell. The PIIb cell divides twice to produce a glial cell, a sheath cell and a neuron [18]–[21]. The glial cell undergoes programmed cell death shortly after its birth [20]. During macrochaete development, each SOP differentiates at different times at different positions of the wing discs. For example, SOPs of single posterior scutellar (pSC) bristles differentiate 30 hours before pupal formation (BPF) [17]. The onset of asymmetric cell division in the pSC cell lineage appears to occur just before pupal formation and end around 3 to 5 hours APF [17]. After cell differentiation, both the shaft cell and the socket cell undergo a few rounds of replication in the case of the microchaete cell lineage [21]–[23]. However, little is known about how much endoreplication occurs in shaft and socket cells in the macrochaete cell lineage.

Why should we focus on the macrochaete bristle system? Since the position of each macrochaete is fixed [24] and division timing of microchaetes is well-characterized, cell lineage analysis is readily available. Therefore in contrast to other endocycling cells, such as the fat body and salivary gland cells it is possible to analyse the dynamics of DNA replication in a particular cell lineage during development. Even in the case of follicle cells, in which mechanisms of endoreplication have been extensively studied, the timing appears not to be synchronized within an egg chamber [10]. Recently, live imaging of a single microchaete cell lineage showed that shaft and socket cells undergo three and two rounds of endoreplication, respectively, between 21 and 36 hours APF at 30°C [25]. However, although the precursors of microchaetes form during a 4–6 hour period [25], [26] in all Diptera, the position of microchaetes is less precisely specified than that of the macrochaetes [26]. This causes problems in the study of particular microchaete cell lineages which do not occur in macrochaetes due to their regular appearance and larger cell size. Therefore dissecting the mechanisms of endoreplication during a particular macrochaete cell lineage will allow further exploration of associated mechanisms of growth and endoreplication during development.

In the present study, we carried out direct measurement analyses and BrdU incorporation assays to determine the timing and frequency of endoreplication during pSC macrochaete development. Our findings revealed several aspects of endoreplication in shaft and socket cells of macrochaete cell lineages.

## Results

### Dynamic change in sizes of shaft and socket cells during pSC bristle development

To understand the dynamics of the endocycle in macrochaete cell lineages, we focused on pSC bristles (Fig. 1Q). These are known to complete cell differentiation at around 5 hours APF [17] but later endocycles remain uncharacterized as yet. In order to understand the development of shaft cells, we first determined the process of nuclear growth in pSC shaft cells from 8 to 29 hours APF. We utilized the reporter line, A101-lacZ line [17] which has been widely employed as a marker line for sensory organ cell lineages. The section area of shaft cell nuclei stained with anti-lacZ antibodies became 49 μm^2^ at 8 hours APF (Fig. 1A–C, P), beginning to rise after 12 hours APF and becoming 69 μm^2^ at 18 hours APF, 83 μm^2^ at 20 hours APF, 120 μm^2^ at 22 hours APF, 129 μm^2^ at 24 hours APF, 178 μm^2^ at 26 hours APF and finally 273 μm^2^ at 29 hours APF (Fig. 1D–P). Statistical analyses showed that there is no significant difference in size of shaft cell nucleus between 8 and 12 hours APF (t-test, *P* = 0.40) and 25 and 26 hours APF (t-test, *P* = 0.08). A slight decrease in nuclear size between 23 and 24 hours APF was observed (t-test, *P>0.05). However, this may be caused by the asynchronous age of pupae, triggered by differences in growing conditions (density etc) and the lower sample number at 23 hours APF (N = 3). All these results suggest that the shaft cell nucleus may start to grow at around 12 hours APF after differentiation and continuously grows afterward.

### Dynamic change in C values of shaft and socket cells during pSC bristle development

**Figure 2:**
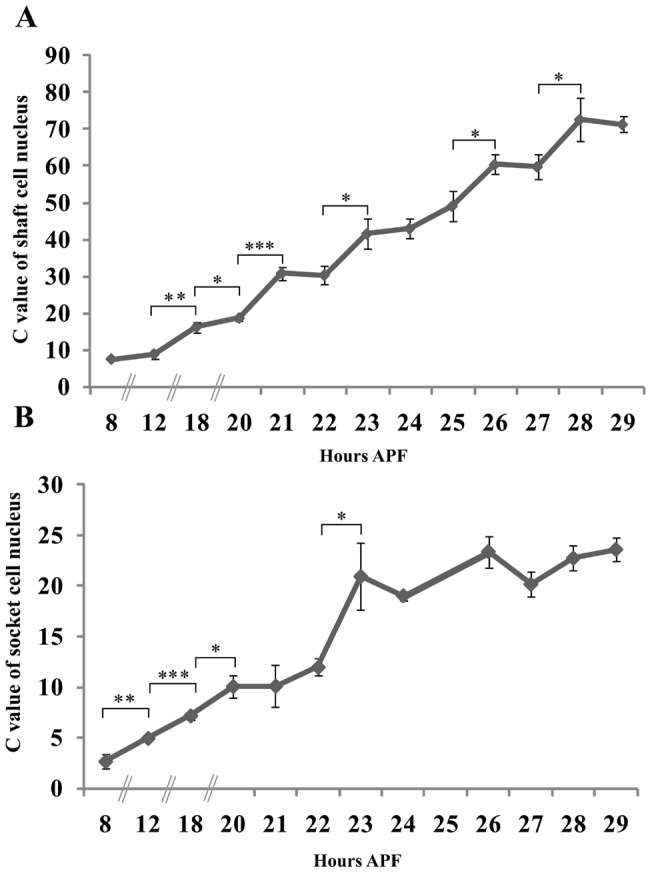
Dynamic changes in the C values of pSC shaft and socket cells from 8 to 29 hours APF. Pupae with the *A101-lacZ /+* genotype were grown till 8, 12, 18, 20, 21, 22, 23, 24, 25, 26, 27, 28, 29 hours APF and dissected thoraxes were stained with anti-lacZ and DAPI. n = 7, 3, 12, 8, 9, 7, 5, 8, 5, 7, 8, 11, 7 or n = 4, 2, 10, 6, 4, 6, 5, 3, 2, 7, 6, 10, 6 corresponds to sample numbers at 8, 12, 18, 20, 21, 22, 23, 24, 25, 26, 27, 28, 29 hours APF for shaft cells (A) or socket cells (B), respectively. The Y axis indicates the C value of each nucleus. Brackets indicate the time points where significant increases in C values were observed. Significant differences in mean values at each time point to those of adjacent time points were set at *P<0.05, **P<0.01 and ***P<0.001.

**Figure 3:**
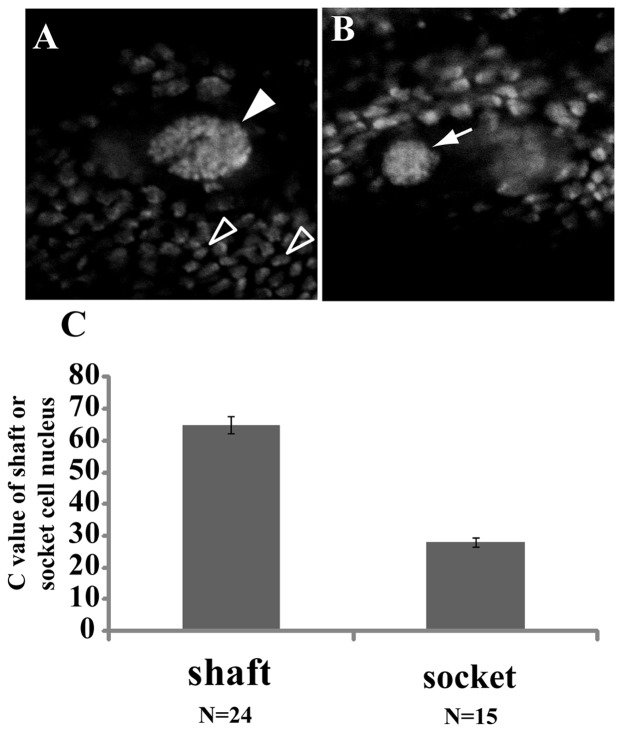
The C values of pSC shaft and socket cells at 29 hours APF as measured with a confocal laser microscope. Wild type female pupae were grown till 29 hours APF and dissected thoraxes were stained with PI. (A) The white arrowhead indicates a shaft cell nucleus. Open arrowheads indicate epidermal cell nuclei. (B) The arrow indicates a socket cell nucleus. (C) Quantification of C values in shaft or socket cell nuclei at 29 hours APF. The Y axis indicates C values.

Next, to determine to what extent the shaft and socket cells undergo endoreplication during development, we carried out DAPI-staining and measured the dynamics of chromatin values (C values) of shaft and socket cell nuclei in pSC bristle cell lineages from 8, 12, 18 hours APF and 20 to 29 hours APF at one hour intervals. The C values are defined as the relative genomic DNA contents of shaft or socket cell nuclei relative to mean values of those of surrounding epidermal cells (see Materials and Methods). The results showed that the C values in shaft cell nuclei were 8C at 8 hours APF and finally increased up to 72C at 29 hours APF (Fig. 2A), a nine-fold increase in genomic DNA content during 21 hours of development. In contrast, the C value in socket cell nuclei was about 4C at 8 hours APF and finally increased up to about 24C at 29 hours APF (Fig. 2B), a six-fold increase. However, relatively large variability was seen in shaft and socket cells at some developmental stages (compare DNA contents at 23 with 24 hours APF). This may be due to the asynchronous age of pupae, influenced by the growing conditions and/or inaccuracy of the imaging method used. The genomic DNA contents were measured by a conventional fluorescent microscope equipped with a cooled CCD camera. This may lead to overlap of genomic DNA with that of neighboring cells in some cases. To further confirm the validity of the measurements, we performed Propidium iodide (PI) staining, and determined the C values of shaft and socket cells by measuring the DNA content of shaft, socket and surrounding epidermal cells at 29 hours APF with a confocal laser microscope (see Materials and Methods). The results showed that the C values of both shaft and socket cells were about 65C and 28C, respectively (Fig. 3A–C). These C values are nearly equal with those measured with DAPI staining utilizing a conventional fluorescent microscope, confirming that the quantification of C values with the conventional microscope is appropriate. Taken together, these results suggest that two rounds of endoreplication occur for shaft and socket cell from 18 to 29 hours APF.

Next, to further explore the detailed change in the C values of shaft and socket cells, we performed statistical analyses and compared the means of the C values at each time point to those of adjacent time points (Fig. 2). Significant increases in shaft cells were observed from 12 to 18 hours APF (t-test, **P<0.01), 18 to 20 hours APF (t-test, *P<0.05), 20 to 21 hours APF (t-test, ***P<0.001), 22 to 23 hours APF (t-test, *P<0.05), 25 to 26 hours APF (t-test, *P<0.05) and 27 to 28 hours APF (t-test, *P<0.05) (Fig. 2A, see arrows). Significant increases in the socket cells were observed from 8 to 12 hours APF (t-test, **P<0.05), 12 to 18 hours APF (t-test, ***P<0.001), 18 to 20 hours APF (t-test, *P<0.05), and 22 to 23 hours APF (t-test, *P<0.05). The results showed that genomic DNA contents in shaft and socket cells increase in a phased manner during restricted periods of its development. The data suggest that DNA replication likely occurred in those periods where an increase in C value was observed.

### Dynamics of endoreplication of shaft and socket cells during pSC bristle development

**Figure 4 pone-0038714-g004:**
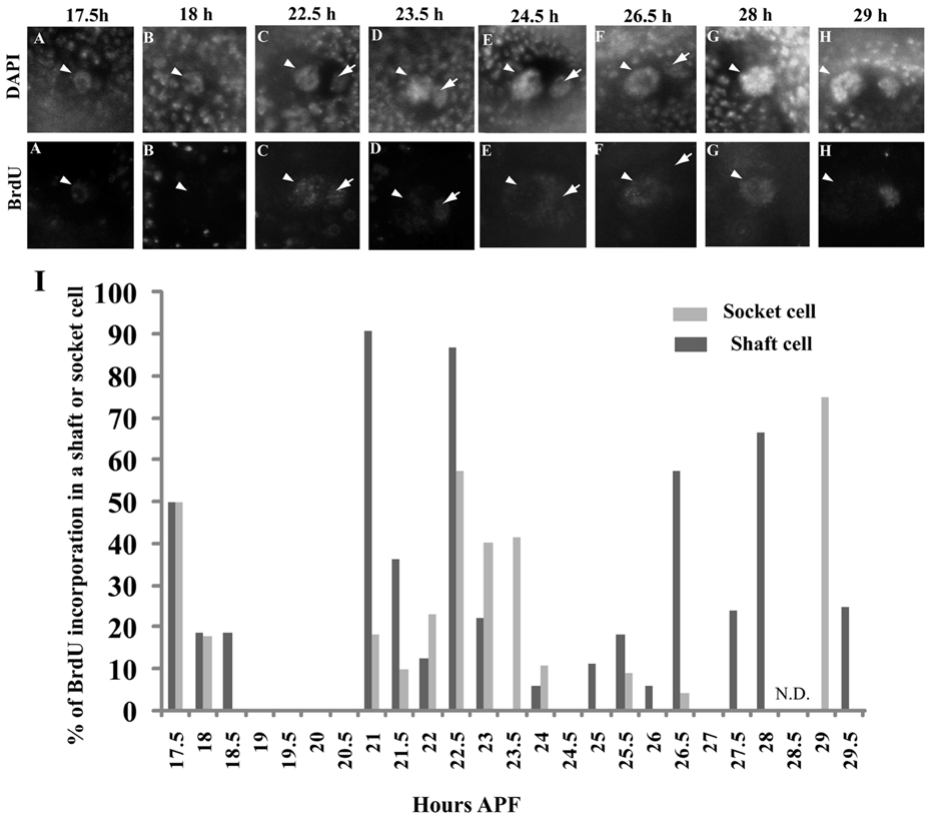
Dynamics of BrdU incorporation in pSC shaft and socket cells from 17.5 to 29 hours APF. Pupae with the +/+ genotype were grown until particular times of development and BrdU incorporation assays were performed as described in the Materials and Methods. Images show pSC shaft and socket cells stained with DAPI and anti-BrdU antibodies (A–H). Arrows indicate socket cell nuclei. Arrowheads indicate shaft cell nuclei. Note the BrdU incorporation observed in A, C, F, G in shaft cells and in C, D, H in socket cells. (I) Pattern of BrdU incorporation in shaft cells from 17.5 to 29 hours APF. The Y axis indicates the % of BrdU incorporation in shaft and socket cells. Sample numbers and detailed data are described in Table S1.

To confirm the validity of the results (Fig. 2), we examined the timing of S phase in pSC shaft and socket cells of a left side of a thorax by carrying out BrdU incorporation assays at 30 minute intervals from 17.5 to 29 hours APF (Note that N.D. at 28.5 hours APF) (see Materials and Methods). In this study, S phase is defined as the period where more than 50% of shaft or socket cells show BrdU incorporation. It is difficult to obtain 100% BrdU staining for a variety of reasons such as the asynchronous stage of the pupae as stated earlier. Based on 50% BrdU staining in shaft cells S phases were observed at 17.5, 21, 22.5, 26.5 and 28 hours APF (Figs. 4, Table S1) (in detail, 50% for 17.5, 91% for 21, 87% for 22.5, 57% for 26.5 and 67% for 28 hours APF). The timing of BrdU incorporation was generally consistent with times when statistically significant changes in the C value of shaft cells were observed (Fig. 2A, see brackets, an exception is the period of 18 to 20 hours APF). In contrast, in the case of socket cells, S phases were observed at 17.5, 22.5 and 29 hours APF (Figs. 4, Table S1). The timing again was largely consistent with times when statistically significant changes in the C value of socket cells were observed (Fig. 2B, see arrows, an exception is the period of 28 to 29 hours APF). However, slight BrdU incorporation signals were also observed between 21.5 and 22 hours APF and between 24 and 26 hours APF where no significant increase in the amount of DNA is seen (Fig. 2 and 4I). This again may be due to the asynchronous stage of the pupae, stated earlier. However, we cannot exclude the possibility that these signals reflect the length of individual S phases as S phase lengths in shaft and socket cells of microchaetes vary between 187 and 291 minutes at 30°C [23]. All these results suggest that S phase in pSC shaft cells may occur at around 17.5, 21, 22.5, around 26.5 and 28 hours APF in pSC shaft cells, whereas in pSC socket cells it may occur at 17.5, around 22.5 and 29 hours APF.

### Endoreplication of socket cell may occur twice at around 23 and 29 hours APF

The results of two different experiments suggest that endoreplication in pSC socket cell occurs twice (Fig. 2, 3, 4). However endoreplication in pSC shaft cell appears to occur twice (Fig. 2 and 3) or four times (Fig. 4). To further explore validity of the results, we performed statistical tests and compared actually measured C values with theoretical C values in shaft and socket cells at a given period of times. Based on results of the BrdU assays (Fig. 4), we defined the theoretical values according to the timing of possible S phase (see Materials and Methods).

For shaft cells, the two theoretical curves are very similar to the actual measured curve until 22 hours APF (an exception is 20 hours APF) (Fig. S1A). However, the actual measured curve subsequently became far different from those of the two theoretical curves (theoretical values 1: t-test, *P<0.05 for 25, 28, 29 hours APF, **P<0.01 for 24 hours APF, ***P<0.001 for 26 hours APF) (theoretical values 2: t-test, *P<0.05 for 25 hours APF, **P<0.01 for 23 hours APF, ***P<0.001 for 24, 27, 28, 29 hours APF). Interestingly, the actual measured C values and the theoretical values both ended up around 64C. These results suggest that the measurement of the DNA content of shaft cells (Fig. 2, 3) is appropriate for the model (theoretical values 1). It further suggests that complete endoreplication might occur twice at around 21 and 27 hours APF. However, we cannot exclude the possibility that the type of endoreplication changed around 22 hours APF and incomplete DNA replication may occur four times during development.

For socket cells, the results showed that the theoretical curve appears to be similar to actually measured curve (Fig. S1A). However there are several significant differences at some time points (t-test, *P<0.05 for 18, 22, 27 hours APF, **P<0.01 for 26 and 28 hours APF, ***P<0.001 for 29 hours APF). Considering that the C values of socket cells at 29 hours APF end up around 28 C (Fig. 3), these results suggest that complete endoreplication may occur twice at around 23 and 29 hours APF in socket cells.

### Endocycling may be synchronized in the same types of macrochaetes

**Figure 5:**
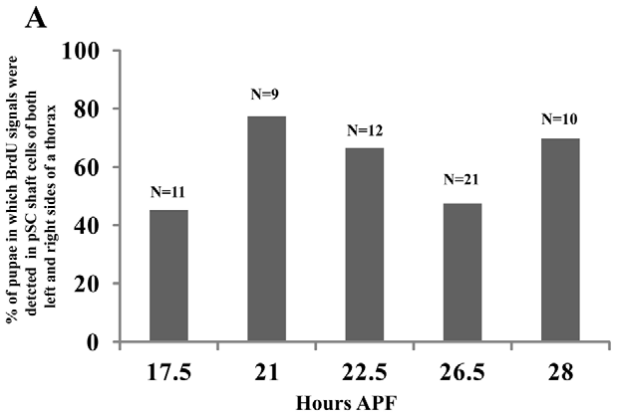
The endocycle is synchronized in pSC macrochaetes. Wild type pupae were grown until particular times of development and BrdU incorporation assays were performed as described in the Materials and Methods. The Y axis indicates % of pupae in which BrdU signals were simultaneously detected in pSC shaft cells of both left and right sides of a thorax. Sample numbers are shown at the top of each bar.

The patterns of BrdU incorporation in pSC shaft and socket cells appear to be almost identical in both the left and right side of the thorax (Table S1), suggesting that endocycling is synchronized in pSC macrochaetes. To further confirm the hypothesis, we reanalyzed the pre-existing data (Fig. 4 and Table S1) and counted the pSC shaft cells that are simultaneously BrdU-positive on both left and right sides of an adult thorax at 17.5, 21, 22.5, 26.5 and 29 hours APF. High ratios of pSC shaft cells that are simultaneously BrdU-positive on both sides were observed at several stages of development (Fig. 5) (in detail, 46% at 17.5, 78% at 21, 67% at 22.5, 48% at 26.5 and 70% at 28 hours APF). The results strongly support the hypothesis that endocycling is synchronized in pSC macrochaetes.

**Figure 6:**
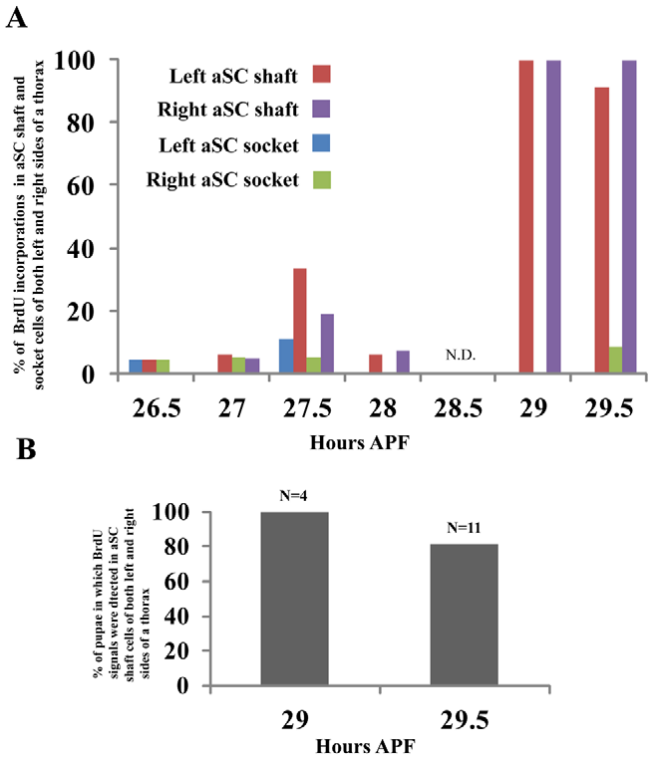
Dynamics of endoreplication in aSC shaft and socket cells from 26.5 to 29 hours APF. (A) Dynamics of BrdU incorporation in aSC shaft and socket cells from 26.5 to 29 hours APF. Wild type pupae were grown until 26.5, 27, 27.5, 28, 29, 29.5 hours APF and BrdU assays were carried out. BrdU signals were counted in shaft and socket cells of aSC bristle lineages in both the left and right sides of a thorax. The Y axis indicates the % of BrdU incorporation in shaft and socket cells in left and right sides of a thorax. n = 23, 17, 18, 17, 4, 11 or n = , 23, 21, 21, 14, 5, 10 or n = 23, 16, 18, 16, 5, 11 or n = 23, 19, 20, 14, 5, 12 corresponds to sample numbers at 26.5, 27, 27.5, 28, 29, 29.5 hours APF for left aSC shaft, right aSC shaft, left aSC socket cells or right aSC socket cells, respectively. (B) Endoreplication is synchronized in aSC shaft cells. The Y axis indicates % of pupae in which BrdU signals were simultaneously detected in aSC shaft cells of both left and right sides of a thorax.

To examine whether endocycling is synchronized in other types of macrochaetes, we examined the timing of S phase in the left and right sides of aSC shaft and socket cells by carrying out BrdU incorporation assays at 30 minute intervals from 26.5 to 29 hours APF (Note that N.D. at 28.5 hours APF). The results showed that 90% of BrdU incorporations were observed at 29 and 29.5 hours APF in shaft cells of both the left and right side of the thorax (Fig. 6A). We also found that the timings of the endoreplication at these time points are almost the same in both left and right sides of an adult thorax (Fig. 6B). These results support the idea that endocycling in shaft cells is synchronized in specific types of macrochaetes.

## Discussion

### Dynamics of endoreplication in pSC bristle cell lineages

**Figure 7:**
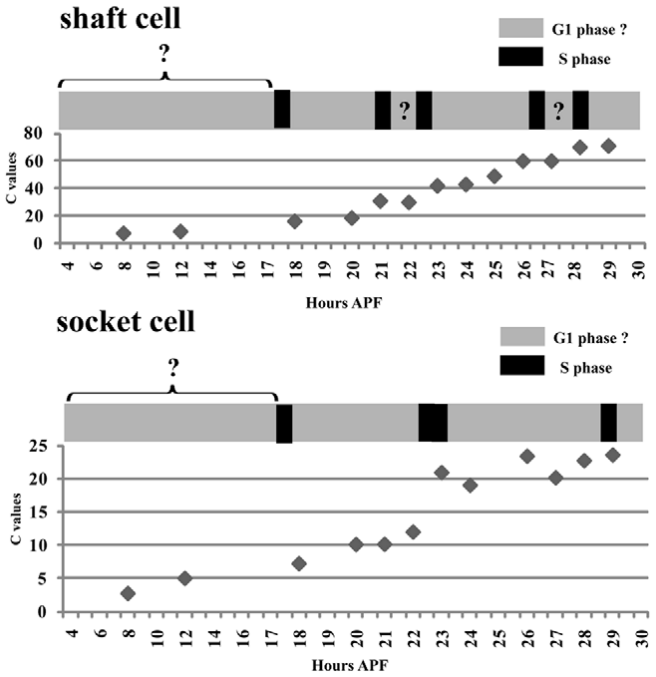
Model of dynamics of endoreplication during the development of pSC shaft and socket cells. Pattern of endoreplication and dynamic changes in C values in shaft and socket cells from 4 to 30 hours APF. Black boxes indicate S phases. Gray boxes indicate possible G1 phases. Patterns of BrdU incorporation in the periods indicated by Gray boxes with question marks were not determined.

Our quantitative analyses provide the first demonstration of the dynamics of endoreplication during development of a single macrochaete (Fig. 4, see model in Fig. 7). We developed simple and quantitative methods to determine dynamic change in sizes and C values in shaft and socket cells (Fig. 1, 2). In combination with BrdU incorporation assays, a series of experiments demonstrated the following. (1) Endoreplication in shaft cells occurs at a higher frequency than that in socket cells (Fig. 3, 4, 7 and Table S1); (2) Timing of endoreplication in shaft cells differs from that in socket cells (Fig. 4, 7 and S1); (3) Endocycling in macrochaetes is synchronized (Fig. 4, 5, 6 and S1); (4) Patterns of endocycling differ, depending on the type of macrochaete (Fig. 4, 5, 6 and S1). These findings should contribute to a deeper understanding of the mechanisms of endocycling in macrochaete cell lineages.

### Frequencies of endoreplication in pSC shaft and socket cells

Our findings suggest that pSC socket cells undergo endoreplication at around 23 and 29 hours APF (Fig. 3–5). In contrast, we could not fully elucidate endoreplication in pSC shaft cell. Our data suggests two possibilities for the pattern and frequency of endoreplication in pSC shaft cells. The first is that the shaft cells undergo four rounds of endoreplication at 21, 22.5, 26.5, 28 hours APF. The second is that the shaft cells undergo two rounds of endoreplication at around 21 and 26.5 hours APF. The former model is supported by the BrdU assay data (Fig. 4), but not by the actual measured C values (Fig. 3 and S1). In contrast, the latter model appears to be more consistent with the actual measured C values (Fig. 3 and S1), but not with the BrdU assay data (Fig. 4). However, when measuring BrdU incorporation, our analyses excluded late S phase time points when the replication of chromocenters, the principle sites of aggregated heterochromatin [10], [23], is observed. In addition to this, it has been demonstrated recently that shaft and socket cells have an early and late S phase with durations of up to 155 and 136 minutes, respectively [23]. Although the experiments were carried out at 30°C and cannot be directly compared, these data imply that one S phase in pSC shaft cells may be as long as that in microchaete shaft cells, and two distinct BrdU incorporation signals which are 21 and 22.5 or 26.5 and 28 hours APF may belong to the same S phase. If this were the case then it would favor the model involving two rounds of endoreplication. However, we found that there are significant differences at many stages of development, especially in the period of 23 to 26 hours APF (Fig. S1A, compare actually measured values with theoretical curve 1 in shaft cells). Therefore, we cannot exclude the possibility that the mechanism of endoreplication in the shaft cells changes from 22 hours APF. In order to clarify which models are more plausible, investigation of the duration of G and S phases by the recently developed in vivo live imaging system [23], [27] in shaft as well as socket cells may be required.

### Perspective

Our results clarify the dynamics of endoreplication at later time points in shaft and socket cells. However in order to fully characterize the replication cycles in these cells the dynamics of endoreplication from the end of cell differentiation through asymmetric cell division to 8 hours APF has to be clarified (Fig. 4). Here we found the C value in shaft cells at 8 hours APF to be about 8C (Fig. 2A). As reported earlier, shaft and socket cells of pSC macrochaete differentiate during 3 to 5 hours APF [17]. Therefore, it is possible that at least two rounds of endoreplication may occur during this period. In the case of follicle cells, genomic DNA is fully replicated during the first three rounds of endoreplication [10] then, particular genomic regions are amplified during and after [28]. Therefore it would be interesting to ask whether a similar mechanism operates in shaft cells so that genomic DNA is fully replicated at each S phase until 18 hours APF, and then further increases only occur in particular genomic regions. It would also be interesting to determine whether the mitotic to endocycle transition, mostly studied in follicle cells [29], occurs or not in shaft and socket cells.

## Materials and Methods

No specific permits were required for the described field studies. The present studies did not involve endangered or protected species.

### Fly strains

Canton S was used as the wild type line. The A101-lacZ line was from the Bloomington Stock Center.

### Collection of prepupae and raising pupae

We collected 150 to 200 virgin female and male flies and put them in a 200 ml glass bottle with fly food. The mated female flies were allowed to lay eggs for 2 days at 25°C. The white prepupae were collected with a soft ink brush. They were transferred to a plastic tube with a cut paper towel with a small amount of PBS and incubated at 25°C until the appropriate developmental stage. For collection of pupae, sickly pupae with different shapes and color were removed from the samples.

### Immunohistology

Dissected larvae or pupae were fixed in 4% paraformaldehyde (PFA) for 30 minutes. After washing with PBS containing 0.3% Triton X-100 (PBS-T), the samples were incubated with mouse anti-lacZ (Developmental Studies Hybridoma Bank, DSHB, 1∶500) primary antibodies for 2 hours at 25°C;. After washing with PBS-T, samples were incubated with anti-mouse secondary antibodies conjugated with Alexa 594 (Molecular Probe, 1∶600) for 2 hours at 25°C. After washing, the samples were mounted with Vectashield mounting medium containing DAPI (H-1200, Vector laboratories). Images were obtained using an Olympus BX-50 fluorescent microscope equipped with a cooled CCD camera (ORCA-ER; Hamamatsu photonics, Japan) and analyzed with AQUACOSMOS Ver.2.5 software (Hamamatsu photonics).

### BrdU incorporation

For BrdU incorporation experiments, dissected thoraxes were incubated with Grace's medium containing 100 μM of BrdU (5′-Bromo-2′-deoxyuridine Labeling & Detection Kit, Roche) at 25°C for 30 minutes on a shaker and then washed with PBS. After fixing with 4% PFA for 20 to 30 minutes, the samples were incubated with 2N HCl for 15 minutes and neutralized with 0.1 mol/l Na_2_B_4_O_7_ for 5 minutes. After washing with PBS-T several times, the samples were incubated with mouse anti-BrdU IgG (5′-Bromo-2′-deoxyuridine Labeling & Detection Kit, Roche, 1∶25 dilution) on a shaker for 2 to 3 hours at 25°C. After washing with PBS-T, the samples were incubated with the secondary antibody for 2 hours at 25°C.

For calculations of BrdU-positive cells among shaft and socket cells, we focused on shaft and socket cells in a pSC bristle in a left or a right side of an adult thorax (Fig. 1Q) and counted the numbers of BrdU-positive cells. Percentages of BrdU incorporations (Fig. 4) were obtained by dividing the number by the total number of the observations. Shaft and socket cells were identified by size, shape and location as previously described [30].

### Measurement of relative DNA contents of nuclei in shaft and socket cells of pSC bristles

Female pupae were raised at 25°C until the appropriate stage as previously described [27]. Dissected thoraxes were stained with mouse anti-lacZ (DSHB, 1∶500) for 16 hours at 4°C and anti-mouse IgG conjugated with Alexa 594 (Molecular Probe, 1∶600) for 2 hours at 25°C. After incubation in Vectashield mounting medium containing DAPI (Vector laboratories) overnight, images were obtained under the same exposure time. For estimation of relative DNA contents, referred to as C values, the DAPI staining intensities of both pSC shaft and socket cell nuclei as well as three to five clear epidermal cells in the same image were measured using AQUACOSMOS Ver.2.5 soft ware (Hamamatsu photonics). C values were obtained by dividing a value of the intensity of the shaft or socket cell nucleus by the mean value for intensity in epidermal cells.

### Propidium iodide staining

For PI staining, pupae were collected at 29 hours APF. Dissected pupae were fixed in 4% PFA for 20 minutes. After washing with PBS-T twice, the samples were incubated with 20 μg/ml RNase A and 14 μg/ml PI for 30 minutes at 25°C. After washing with PBS-T once and PBS twice for 10 minutes each, the samples were mounted with Vectashield (H-1000, Vector laboratories). Images were obtained using an Olympus FLUOVIEW FV1000 confocal laser microscope and analyzed with FV10-ASW software [Ver1.7] (Olympus). For estimation of the relative DNA contents, the PI intensities of both pSC shaft and socket cell nuclei as well as five clear epidermal cells in the same image were measured using the FV10-ASW software (Olympus). C values were obtained by dividing a value of the PI intensity of the shaft or socket cell nucleus by the mean value for PI intensity in epidermal cells.

### Comparisons of actually measured C values with theoretical C values in a shaft or a socket cell

Theoretical values were set according to times of possible S phase, observed in the results of BrdU incorporation assays (Fig. 4). The theoretical values of 1 or 2 for shaft cells are defined as the point where complete endoreplication occurs twice at 21 and 27 hours APF, or four times at 21, 23, 27 and 29 hours APF, respectively. The theoretical value 1 for socket cells is defined as the point where complete endoreplication occurs at 23 and 29 hours APF. T-tests were performed between the theoretical values and actual values at 18, 20, 21, 22, 22, 23, 24, 25, 26, 27, 28, 29 hours APF in a shaft or a socket cell. Significance levels were set at *P<0.05, **P<0.01 and ***P<0.001.

### Statistical analysis

All statistical analyses were performed using EXCEL Toukei Ver.6.0 (ESUMI). For comparison between two groups T-tests (Fig. 1, 2 and Fig. S1) were employed. Significance levels for each test were set at *P<0.05, **P<0.01 and ***P<0.001. All data shown are mean ± SEM values.

## Supporting Information

Figure S1 **Comparisons of actually measured C values with theoretical C values in shaft (A) and socket cells (B) of pSC bristles.** Theoretical values were set according to the results of BrdU incorporation assays (Fig. 4). Statistical analyses were performed as described in Material and Methods. Significant differences in mean values were set at *P<0.05, **P<0.01 and ***P<0.001.
(TIF)

Table S1 **Quantitative data of BrdU incorporation assays in left and right pSC macrochaete lineages.** Number of samples and % of BrdU incorporation in both shaft and socket cells in left and right PSC macrochaete cell lineages are described.
(TIF)
