# Supplementary figures and images for: Dynamics of Endoreplication during Drosophila Posterior Scutellar Macrochaete Development
Source: PLoS One. 2012 Jun 6;7(6):e38714. doi: 10.1371/journal.pone.0038714 (PMC3368872; doi:10.1371/journal.pone.0038714)

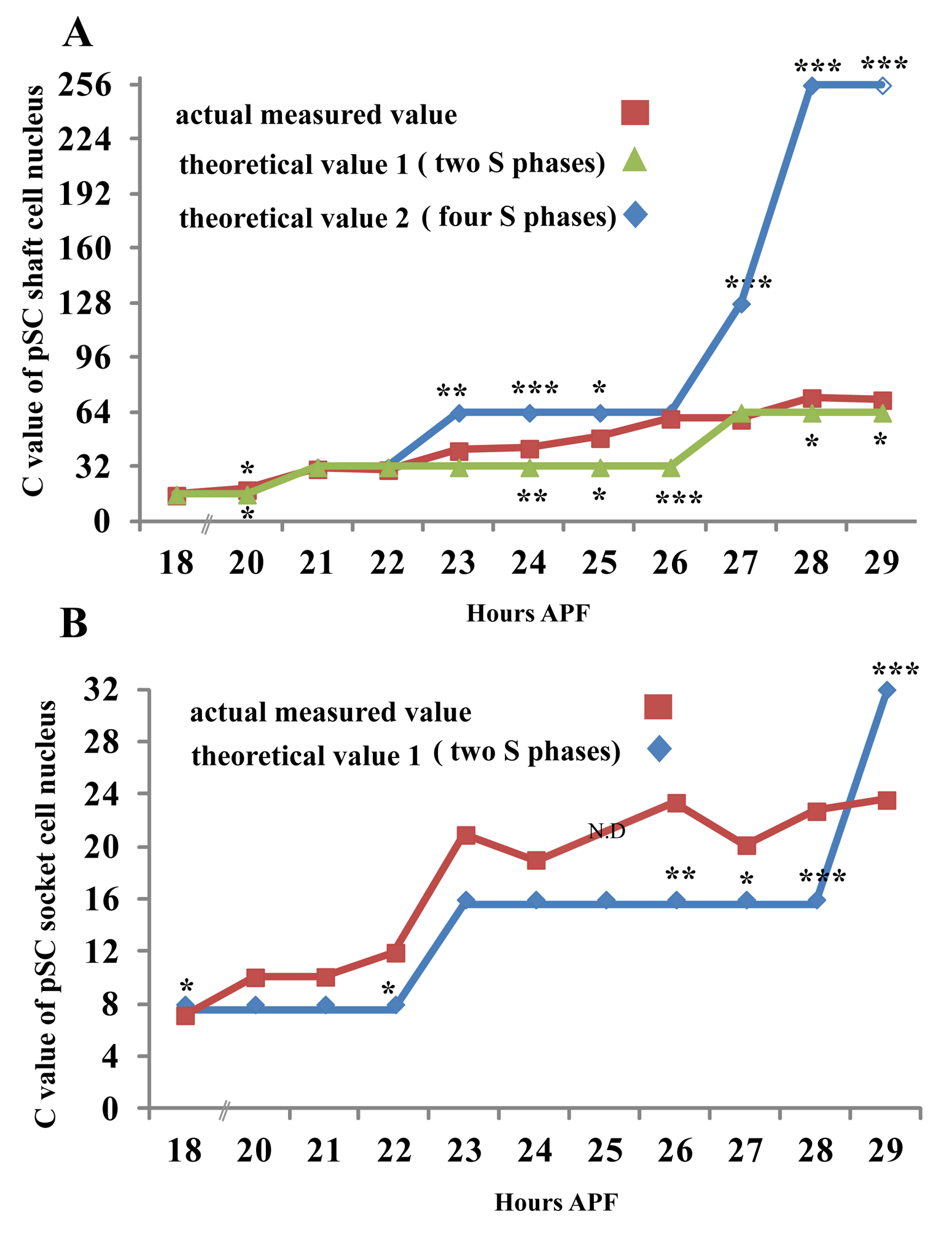

Supplement: Figure S1 — Comparisons of actually measured C values with theoretical C values in shaft (A) and socket cells (B) of pSC bristles. Theoretical values were set according to the results of BrdU incorporation assays (Fig. 4). Statistical analyses were performed as described in Material and Methods. Significant differences in mean values were set at *P<0.05, **P<0.01 and ***P<0.001. (TIF) [file pone.0038714.s001.tif]

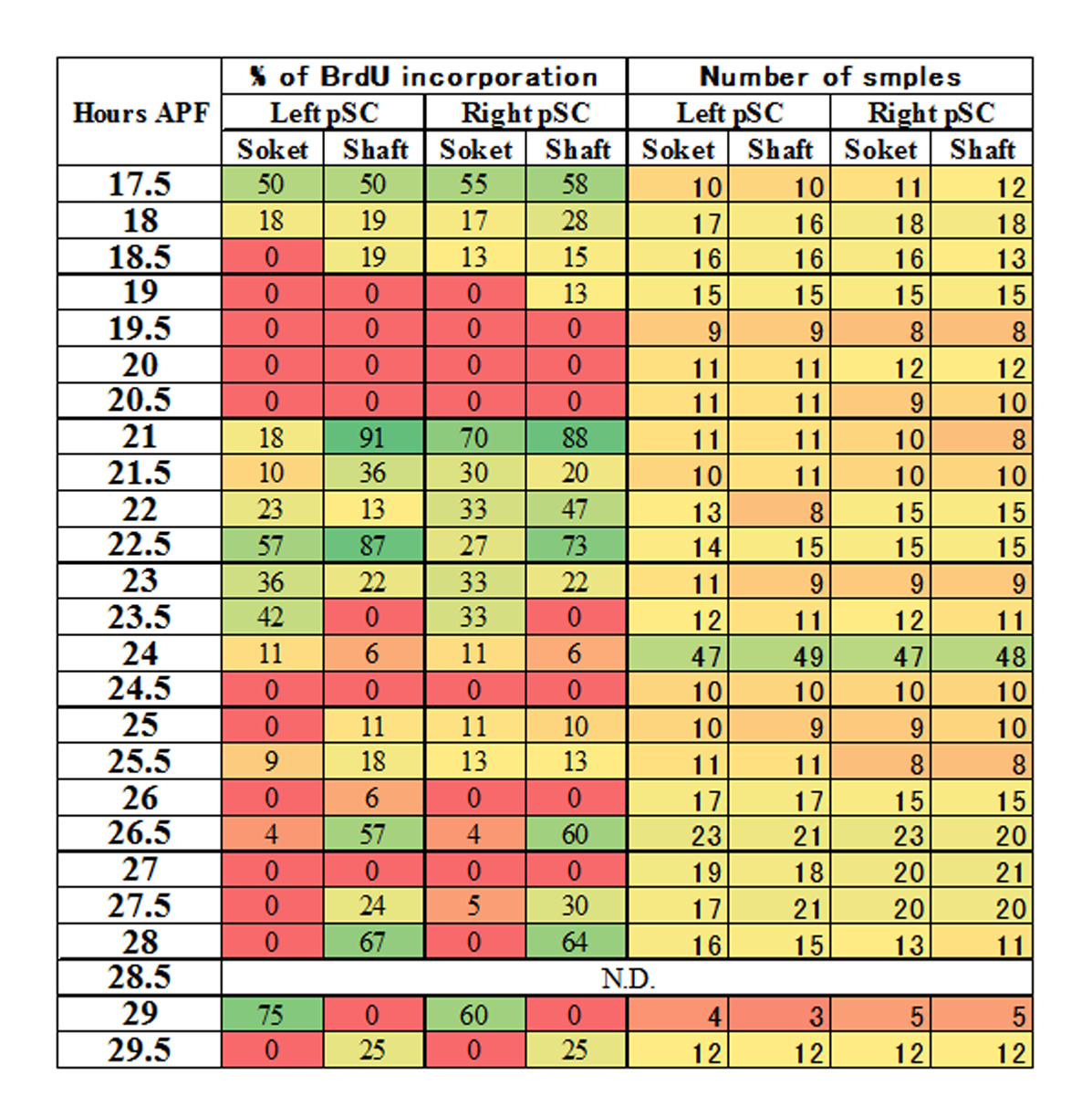

Supplement: Table S1 — Quantitative data of BrdU incorporation assays in left and right pSC macrochaete lineages. Number of samples and % of BrdU incorporation in both shaft and socket cells in left and right PSC macrochaete cell lineages are described. (TIF) [file pone.0038714.s002.tif]
